# Placental Size Is Associated Differentially With Postnatal Bone Size and Density

**DOI:** 10.1002/jbmr.2840

**Published:** 2016-04-22

**Authors:** Christopher R Holroyd, Clive Osmond, David JP Barker, Sue M Ring, Debbie A Lawlor, Jon H Tobias, George Davey Smith, Cyrus Cooper, Nicholas C Harvey

**Affiliations:** ^1^ MRC Lifecourse Epidemiology UnitUniversity of SouthamptonSouthamptonUK; ^2^ MRC Integrative Epidemiology UnitUniversity of BristolBristolUK; ^3^ School of Social and Community MedicineUniversity of BristolBristolUK; ^4^ Academic RheumatologyMusculoskeletal Research UnitAvon Orthopaedic CentreBristolUK; ^5^ NIHR Southampton Biomedical Research CentreUniversity of Southampton and University Hospital Southampton NHS Foundation TrustSouthamptonUK; ^6^ NIHR Musculoskeletal Biomedical Research UnitUniversity of Oxford, Nuffield Orthopaedic CentreHeadingtonOxfordUK

**Keywords:** OSTEOPOROSIS, EPIDEMIOLOGY, PLACENTA, BONE MASS, DXA, pQCT, ASLPAC

## Abstract

We investigated relationships between placental size and offspring adolescent bone indices using a population‐based, mother–offspring cohort. The Avon Longitudinal Study of Parents and Children (ALSPAC) recruited pregnant women from the southwest of England between 1991 and 1993. There were 12,942 singleton babies born at term who survived at least the first 12 months. From these, 8933 placentas were preserved in formaldehyde, with maternal permission for their use in research studies. At the approximate age of 15.5 years, the children underwent a dual‐energy X‐ray absorptiometry (DXA) scan (measurements taken of the whole body minus head bone area [BA], bone mineral content [BMC], and areal bone mineral density [aBMD]). A peripheral quantitative computed tomography (pQCT) scan (Stratec XCT2000L; Stratec, Pforzheim, Germany) at the 50% tibial site was performed at this visit and at approximately age 17.7 years. In 2010 a sample of 1680 placentas were measured and photographed. To enable comparison of effect size across different variables, predictor and outcome variables were standardized to *Z*‐scores and therefore results may be interpreted as partial correlation coefficients. Complete placental, DXA, and pQCT data were available for 518 children at age 15.5 years. After adjustment for gender, gestational age at birth, and age at time of pQCT, the placental area was positively associated with endosteal circumference (β [95% CI]: 0.21 [0.13, 0.30], *p* < 0.001), periosteal circumference (β [95% CI]: 0.19 [0.10, 0.27], *p* < 0.001), and cortical area (β [95% CI]: 0.10 [0.01, 0.18], *p* = 0.03), and was negatively associated with cortical density (β [95% CI]: –0.11 [–0.20, –0.03], *p* = 0.01) at age 15.5 years. Similar relationships were observed for placental volume, and after adjustment for additional maternal and offspring covariates. These results suggest that previously observed associations between placental size and offspring bone development persist into older childhood, even during puberty, and that placental size is differentially related to bone size and volumetric density. © 2016 The Authors. *Journal of Bone and Mineral Research* Published by Wiley Periodicals, Inc. on behalf of American Society for Bone and Mineral Research (ASBMR).

## Introduction

The size of the placenta reflects its ability to transfer nutrients^1^ to the developing fetus, and indices of placental morphology are subject to wide variations.^2^ There is increasing evidence that attributes such as placental area and volume may predict the risk of common chronic noncommunicable diseases (NCDs) in later life. For example, low placental weight at birth has been associated with increased risk of hypertension and coronary heart disease in adulthood.^3^, ^4^ Osteoporosis constitutes a further important NCD and is a major public health concern because of its association with age‐related fragility fractures. We have previously shown that birth weight is associated with bone mineral content (BMC) in adulthood, and that poor early postnatal growth predicts adverse proximal femoral morphology^5^, ^6^ and increased risk of hip fracture in older age.^7^, ^8^ Although placental transfer of nutrition from mother to fetus is critical in the determination of birth weight,^9^ there is a paucity of evidence relating to associations between placental morphology and offspring bone mass. Recently, using data from a large prospective mother–offspring cohort, the Southampton Women's Survey, we observed that placental volume, measured by high‐resolution ultrasound in midpregnancy, was positively associated with neonatal bone size and content measured by dual‐energy X‐ray absorptiometry (DXA).^10^ It remains unclear, however, whether these associations might persist into later childhood and whether placental size may have differential relationships with bone size and density. The aim of this study, therefore, was to investigate whether placental size is associated with indices of bone size, geometry, and density in the offspring, assessed using peripheral quantitative computed tomography (pQCT) during adolescence, in a UK population‐based, mother–offspring cohort.

## Subjects and Methods

### The Avon Longitudinal Study of Parents and Children (ALSPAC)

ALSPAC is a large prospective birth cohort study, the aim of which is the investigation of genetic and environmental influences on childhood health and development. Details of ALSPAC have been published previously^11^, ^12^ but, in brief, all pregnant women living in the former county of Avon in the United Kingdom with an expected delivery date between April 1, 1991 and December 31, 1992 were eligible to take part. During early pregnancy, 14,541 women were recruited (80% to 90% of the target population). Information from early pregnancy onward was collected from a variety of sources including self‐completed questionnaires, medical records, and annual examination and assessment of the children at dedicated research clinics. The study website contains details of all the data that are available through a fully searchable data dictionary (http://www.bris.ac.uk/alspac/researchers/data-access/data-dictionary/).

### Placental and birth measurements

**Figure 1:**
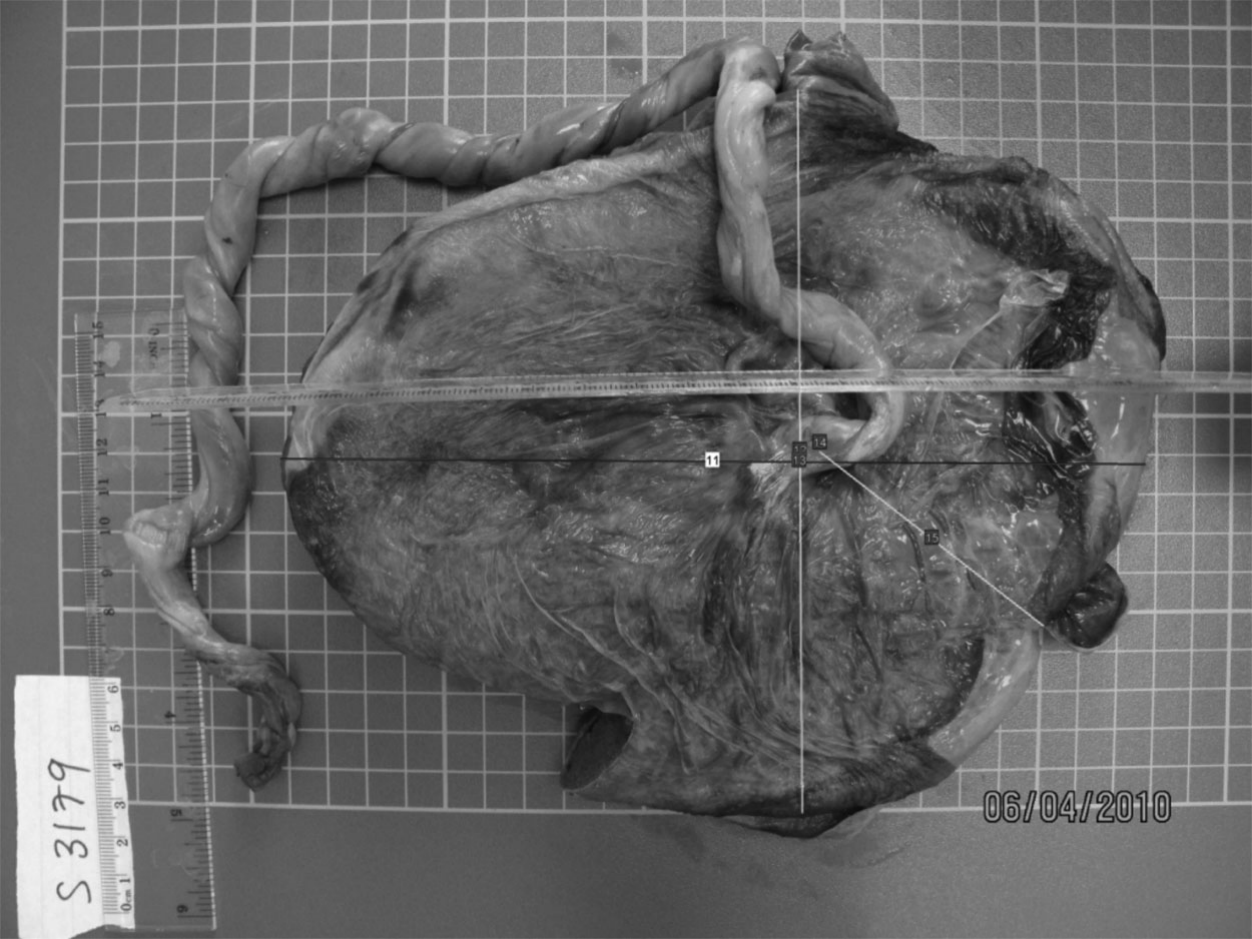
Image of the fetal side of a placenta and umbilical cord. Lines illustrate measurements of length (dark lines) and width (light lines).

There were 12,942 singleton infants born at term (≥37 completed weeks). Premature infants were excluded because prematurity is known to affect skeletal development.^13^ Length of gestation was estimated from the date of the mother's last menstrual period. Birth weights were extracted from hospital records, and birth lengths (crown to heel) were measured using a Harpenden neonatometer (Holtain Ltd., Crymlych, Wales, UK) by ALSPAC staff who visited all study participants within a day after birth. At delivery, the placenta was collected and stored in 10% formaldehyde for later assessment. In 2010 a sample of 1680 placentas, all from one maternity hospital and taken in the order in which they were stored, were removed from their containers, trimmed as per a standard protocol, and measured.^14^ Direct measurements were made of placental thickness, volume, and weight. Both sides of the placenta (maternal and fetal) were then photographed using a digital camera. Each photograph included a ruler to measure the length and breadth of the surface (Fig. 1). Length was defined as the maximal diameter, and breadth was measured at 90 degrees to the midpoint of the length. To calculate area, the placenta was assumed to be elliptical in shape, and area was defined as the product of length and breadth multiplied by π/4. Maximum thickness was measured using a calibrated needle, and volume was estimated as the product of area and maximum thickness.

### Skeletal assessment at ages 9.9, 15.5, and 17.7 years

At age 9 years, children were invited to attend the Focus@9 Research Clinic (Bristol) where whole‐body DXA assessment of bone mass by DXA (Lunar Prodigy; GE Lunar, Madison, WI, USA) was undertaken. At approximately age 15.5 years, all children within the ALSPAC cohort were invited to attend a research clinic as part of a study investigating the effects of physical activity on cortical bone.^15^ Height (to the nearest 0.1 cm) and weight (to the nearest 50 g) were measured using a Harpenden Stadiometer (Holtain Ltd.) and Tanita Body Fat Analyzer (Tanita UK Ltd., Uxbridge, UK), respectively. Measurements were then made of whole‐body bone area (BA), BMC, and areal bone mineral density (aBMD) using a DXA scanner with specific pediatric software (GE Lunar). The children also underwent pQCT assessment of their middle (50% from the distal endplate) right tibia using a Stratec XCT2000L instrument (Stratec, Pforzheim, Germany). Cortical BMD (BMD_C_) and cortical BMC (BMC_C_) were obtained. Periosteal circumference (PC), endosteal circumference (EC), and cortical thickness (Ct.Th) were derived using a circular ring model. Cortical bone was defined using a threshold above 650 mg/cm^3^, as previously described.^15^ The within subject coefficients of variation (CV) for pQCT measurements are displayed in parentheses: tibial length (4.04%), BMC_C_ (2.71%), BMD_C_ (1.29%), PC (1.58%), and EC (4.03%). All scans were reviewed and those with artefact were excluded from analysis. Whole‐body DXA and tibial pQCT assessments were repeated in the cohort at approximately age 17.7 years using identical methods.

### Pubertal assessment

Questionnaires that addressed maturation were mailed to participants at age 13.5 years, permitting ranking of participants according to age of pubertal onset. The puberty questionnaire, known to participants as the Growing and Changing Questionnaire, could be answered by the child, either parent, a guardian, or any combination of these individuals; the participants recorded who completed the questionnaire. The respondent was asked to examine line drawings representing the five Tanner stages for pubic hair and to record which drawing most closely represented the child's current stage of development.

Ethical approval was obtained from the ALSPAC Law and Ethics Committee, and the local research ethics committees. Parental written informed consent and child's assent were obtained for all measurements made.

### Statistical analysis

All variables were checked for normality. Gender differences between baseline characteristics were compared using unpaired *t* tests and chi‐square tests. Pubertal stage information was missing for 42.6% of individuals and in these cases data were imputed: Individuals who did not have pubertal stage information were assigned a value of 4.5, which was close to the mean value (4.46) and stands midway between the two most commonly observed Tanner stages—4 and 5. A sensitivity analysis was undertaken using the complete case data. Univariate and multivariate linear regression were used to relate placental measurements to offspring DXA and pQCT measurements. Although not necessarily true confounders, gestational age at birth is strongly associated with placental size, and offspring age and gender are strongly associated with the bone outcomes: We therefore included these as covariates in all models in order to increase the precision of our estimates. We hypothesized that maternal factors, which have previously been associated with offspring bone mass and body composition, might act through placental size and thus incorporated these into a second model. Puberty has a marked impact on bone development and in a third model we included the child's pubertal stage at age 13.5 years to investigate whether relationships might be mediated via altered timing of pubertal transition. Finally, in order to assess the contribution of the child's current body size, in a further model we additionally included the child's height and weight at the relevant assessment. The covariates included in the four models are:

Model 1: Child's gestational age at delivery, age at pQCT, and gender

Model 2: Same as Model 1 and maternal age, height, weight, and parity at delivery

Model 3: Same as Model 2 and child's pubertal stage at age 13.5 years

Model 4: Same as Model 3 and child's height and weight at the relevant follow‐up assessment

In order to assess the contribution of placental size to change in bone outcomes, we used a conditional multiple regression analysis in which the four models were used as before; however, for each pQCT or DXA measure we additionally included the corresponding measure at the previous time point. This effectively yields the association between placental size and the bone measure at a later time point, over and above that explained by the association between placental size and the bone measure at the earlier time point. We undertook separate analyses to assess whether associations might be mediated via birth weight and by including birth weight in Model 1. Placental measurements and gender interactions were examined; nevertheless, these provided little evidence of gender differences and we therefore analyzed boys and girls together. In line with convention, DXA‐derived, whole‐body bone variables were analyzed minus the head. To enable comparison of effect sizes across relationships, all predictor and outcome variables were standardized to *Z*‐scores with a mean of 0 and an SD of 1. Regression coefficients are therefore representative of SD change in outcome per unit SD change in predictor, and may be interpreted as partial correlation coefficients. Analysis was performed using SPSS Version 21 (IBM UK Ltd., Portsmouth, UK).

## Results

### Baseline characteristics

At age 15.5 years, 5515 children underwent assessment. Of these, 518 (10%; 230 boys and 288 girls) had complete placental, DXA, and pQCT measurements. Table 1 shows the offspring, placental, and maternal characteristics. Offspring DXA indices at ages 9.9, 15.5, and 17.7 years are shown in Supporting Table 1, and participant flow through the study is documented in Supporting Fig. 1. Mean (SD) age for boys and girls was 15.3 (0.2) and 15.4 (0.2) years, respectively. Mean (SD) maternal age at delivery was 29.3 (4.4) years; 50.8% of women were primiparous. At birth, boys were heavier and longer than girls; however, by age 9.9 years there was no difference in height, weight, or any of the DXA variables between the genders. At age 15.5 years boys were taller and heavier, and had higher whole‐body (less head) BA, BMC, and BMD (all *p* < 0.001) than the girls. Similarly, boys had higher cortical area, cortical thickness, cortical content, periosteal circumference and endosteal circumference at the tibial 50% site (all *p* < 0.001); conversely, boys had lower cortical density than girls (*p* < 0.001). Placental measurements did not differ by offspring gender but girls were on average at a greater stage of puberty than boys when assessed at age 13.5 years.

**Table 1:** Baseline Characteristics of Mothers, Placentas, and Children

	WOMEN						
	*n*	Mean (%)	(SD)				
Mothers							
Age (years)	518	29.3	(4.40)				
Height (cm)	504	164.9	(6.6)				
Weight (kg)	492	61.5	(9.9)				
Body mass index (BMI; kg/m^2^)	490	22.6	(3.4)				
Parity							
+Primiparous (Parity = 0)	257	(50.8)					
+Multiparous (Parity ≥1)	249	(49.2)					
	BOYS			GIRLS			
	*n*	Mean/ (%)	(SD)	*n*	Mean	(SD)	*p*
Child							
Birth weight (g)	228	3540.5	553.3	287	3414.4	446.1	0.004
Age at 15.5‐year visit (years)	230	15.3	(0.2)	288	15.4	(0.3)	0.2
Height at 15.5‐year visit (cm)	230	175.0	(8.2)	288	165.0	(5.9)	< 0.001
Weight at 15.5‐year visit (kg)	230	64.1	(12.0)	288	59.1	(9.9)	< 0.001
Gestational age at birth (weeks)	230	39.6	1.6	288	39.7	1.4	0.4
Tanner stage at 13.5 years							
Stage 1	17	(11/8)		11	(5.2)		*p* < 0.001
Stage 2	32	(22.4)		21	(9.9)		
Stage 3	42	(29.4)		50	(23.6)		
Stage 4	41	(28.7)		87	(41.0)		
Stage 5	11	(7.7)		43	(20.3)		
Placental measurements							
Area (cm^2^)	230	286.1	(59.2)	288	284.8	(53.2)	0.8
Volume (cm^3^)	230	793.8	(192.9)	288	797.1	(176.5)	0.8
No. of cotyledons/cm^3^	195	1.7	(0.6)	266	1.8	(0.7)	0.04
Tibial pQCT scan at age 15.5 years							
Cortical area (cm^2^)	230	331.2	(47.7)	288	276.5	(35.8)	< 0.001
Cortical BMD (mg/cm^2^)	230	1076.2	(36.3)	288	1126.2	(24.5)	< 0.001
Cortical thickness (mm)	230	5.7	(0.7)	288	5.3	(0.6)	< 0.001
Cortical content (mg)	230	356.8	(54.6)	288	311.4	(40.4)	< 0.001
Periosteal circumference (mm)	230	76.0	(5.2)	288	69.2	(4.4)	< 0.001
Endosteal circumference (mm)	230	40.2	(5.0)	288	36.2	(4.8)	< 0.001
Tibial pQCT scan at age 17.7 years							
Cortical area (cm^2^)	228	336.6	(51.5)	311	270.5	(34.6)	< 0.001
Cortical BMD (mg/cm^2^)	228	1105.6	(36.6)	311	1133.7	(24.6)	< 0.001
Cortical thickness (mm)	228	5.9	(0.7)	311	5.2	(0.5)	< 0.001
Cortical content (mg)	228	372.5	(59.0)	311	306.7	(39.7)	< 0.001
Periosteal circumference (mm)	228	77.6	(5.2)	311	68.9	(4.5)	< 0.001
Endosteal circumference (mm)	228	40.8	(5.1)	311	36.0	(4.6)	< 0.001

### Placental size and offspring pQCT indices at age 15.5 years

**Figure 2:**
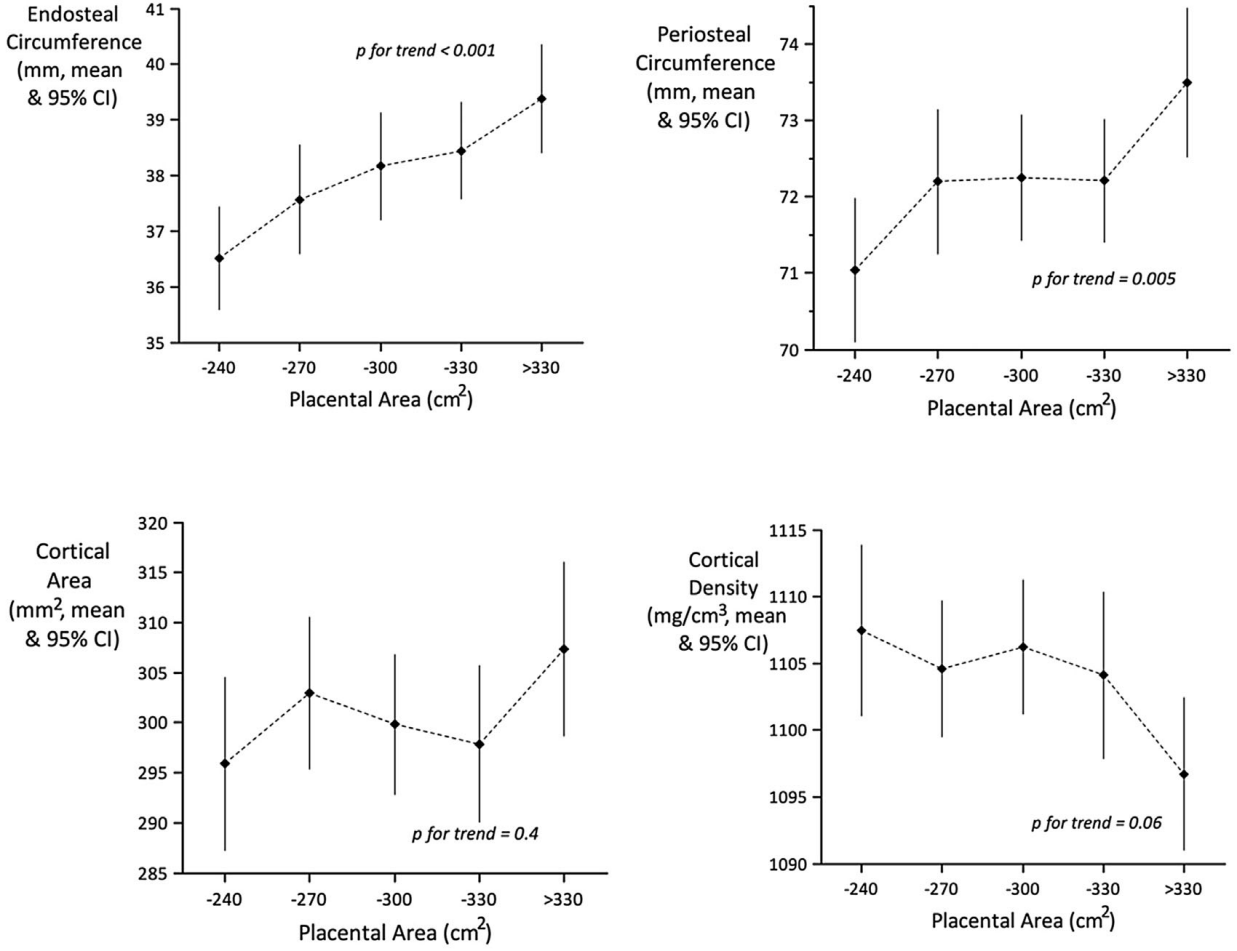
Associations between placental characteristics and childhood pQCT measurements at age 15.5 years. Data are adjusted for child's gestational age at delivery, age at pQCT, gender, and pubertal stage at age 13.5 years. Data also adjusted for maternal age at delivery, maternal height, maternal weight, and parity.

Table 2 summarizes the relationships observed between placental measurements and offspring bone mass. We observed strong positive relationships between placental area and a child's cortical area, periosteal circumference, and endosteal circumference at age 15.5 years, which remained robust after adjusting for gestational age, age at pQCT, and gender (all *p* <0.05). Conversely, there was a negative association between placental area and cortical BMD (β = –0.11 [95% CI: –0.20, –0.03]; *p* = 0.01). These relationships remained, but were attenuated, after additional adjustments for maternal age at delivery, maternal parity, height, and weight, and also after inclusion of the child's pubertal stage at age 13.5 years (except for placental area and cortical area, *p* = 0.06; Table 2; Figure 2). Placental area, adjusted for gestational age, gender, and age at pQCT, was weakly correlated with height at age 15.5 years (*r*_partial_ = 0.07; *p* = 0.048), as was placental volume (*r*_partial_ = 0.1; *p* = 0.03), but adjustment for child's height and weight at age 15.5 years did not materially alter the associations observed. Inclusion of birth weight modestly attenuated the beta coefficients, but the overall pattern of associations remained similar (Supporting Table 2).

**Table 2:** Associations Between Placental Characteristics and Childhood pQCT Measurements at Age 15.5 Years

	Placental measurement															
pQCT at 15.5 years (*n* = 518)	Area (SD)								Volume (SD)							
	B^a^ (95% CI)	*p*	B^b^ (95% CI)	*p*	B^c^ (95% CI)	*p*	B^d^	*p*	B^a^ (95% CI)	*p*	B^b^ (95% CI)	*p*	B^c^ (95% CI)	*p*	B^d^ (95% CI)	*p*
Cortical area (SD)	0.10 (0.01, 0.18)	0.03	0.08 (–0.01, 0.17)	0.07	0.08 (–0.01, 0.17)	0.06	0.01 (–0.06, 0.08)	0.79	0.14 (0.05, 0.23)	0.003	0.09 (–0.02, 0.19)	0.06	0.10 (0.01, 0.19)	0.04	0.06 (–0.02, 0.13)	0.12
Cortical BMD (SD)	–0.11 (–0.20, –0.03)	0.01	–0.14 (–0.22, –0.05)	0.003	–0.13 (–0.22, –0.05)	0.002	–0.16 (–0.24, –0.07)	< 0.001	–0.09 (–0.18, 0.004)	0.06	–0.10 (–0.20, –0.01)	0.04	–0.09 (–0.18, 0.003)	0.06	–0.10 (–0.19, –0.01)	0.03
Cortical thickness (SD)	–0.04 (–0.13, 0.04)	0.36	–0.07 (–0.16, 0.02)	0.14	–0.07 (–0.15, 0.02)	0.14	–0.11 (–0.19, –0.03)	0.01	–0.01 (–0.10, 0.09)	0.90	–0.04 (–0.13, 0.06)	0.46	–0.03 (–0.12, 0.07)	0.55	–0.05 (–0.14, 0.03)	0.22
Cortical content (SD)	0.07 (–0.02, 0.15)	0.13	0.05 (–0.04, 0.13)	0.30	0.05 (–0.04, 0.13)	0.27	–0.03 (–0.09, 0.04)	0.43	0.11 (0.02, 0.20)	0.02	0.06 (–0.03, 0.16)	0.18	0.07 (–0.02, 0.16)	0.12	0.03 (–0.04, 0.10)	0.39
Periosteal circum (SD)	0.19 (0.10, 0.27)	< 0.001	0.18 (0.10, 0.27)	< 0.001	0.18 (0.10, 0.27)	< 0.001	0.11 (0.04, 0.18)	0.002	0.22 (0.13, 0.31)	< 0.001	0.17 (0.08, 0.27)	< 0.001	0.18 (0.08, 0.27)	< 0.001	0.13 (0.06, 0.21)	< 0.001
Endosteal circum (SD)	0.21 (0.13, 0.30)	< 0.001	0.24 (0.15, 0.32)	< 0.001	0.24 (0.15, 0.32)	< 0.001	0.20 (0.12, 0.29)	< 0.001	0.21 (0.12, 0.30)	< 0.001	0.19 (0.10, 0.29)	< 0.001	0.19 (0.10, 0.28)	< 0.001	0.17 (0.08, 0.26)	< 0.001

^a^ Model 1: Adjusted for child's gestational age at delivery, age at pQCT, and gender.

^b^ Model 2: Same as Model 1 and maternal age, height, weight, and parity at delivery.

^c^ Model 3: Same as Model 2 and child's pubertal stage at age 13.5 years.

^d^ Model 4: Same as Model 3 and child's height and weight at age 15.5 years.

The strongest observed relationships were between placental area and measurements of EC and PC (EC: β = 0.21 [95% CI: 0.13, 0.30]; PC: β = 0.19 [95% CI: 0.10, 0.27]; both *p* < 0.001). Similar relationships were observed between placental volume and child pQCT measurements. There was no association between placental size and cortical thickness; a weak association was observed between placental volume and cortical content; however, this relationship was no longer present after maternal and pubertal covariates were incorporated into the regression model. When examined separately by offspring gender, relationships appeared similar in boys and girls, with the *p* value for the interaction placental size*sex on pQCT outcomes > 0.05. In a sensitivity analysis using the complete case data, results were not materially different from those using impute values where pubertal status was missing.

Supporting Table 3 shows that mean placental area and volume did not differ by pubertal stage at age 13.5 years. Supporting Table 4 similarly summarizes the mean pQCT indices (represented as SD scores) by pubertal status at age 13.5 years. Here, there was a trend for greater cortical area, thickness, content, and density with later pubertal stage—both in boys and girls (*p* ≤ 0.01).

**Table 3:** Associations Between Placental Characteristics and Childhood pQCT Measurements at Age 17.7 Years

	Placental measurement															
pQCT at 17.7 years (*n* = 539)	Area (SD)								Volume (SD)							
	B^a^(95% CI)	*p*	B^b^(95% CI)	*p*	B^c^(95% CI)	*p*	B^d^	*p*	B^a^(95% CI)	*p*	B^b^(95% CI)	*p*	B^c^(95% CI)	*p*	B^d^ (95% CI)	*p*
Cortical area (SD)	0.05 (–0.04, 0.13)	0.27	0.05 (–0.04, 0.13)	0.30	0.05 (–0.04, 0.13)	0.30	–0.01 (–0.09, 0.07)	0.82	0.07 (–0.02, 0.16)	0.11	0.05 (–0.04, 0.14)	0.27	0.05 (–0.04, 0.14)	0.26	–0.003 (–0.08, 0.08)	0.94
Cortical BMD (SD)	–0.06 (–0.14, 0.03)	0.18	–0.06 (–0.15, 0.03)	0.18	–0.06 (–0.14, 0.03)	0.17	–0.05 (–0.13, 0.04)	0.26	–0.10 (–0.19, –0.02)	0.02	–0.09 (–0.18, 0.003)	0.06	–0.08 (–0.17, 0.01)	0.08	–0.07 (–0.16, 0.02)	0.14
Cortical thickness (SD)	–0.04 (–0.12, 0.05)	0.39	–0.05 (–0.14, 0.04)	0.27	–0.05 (–0.14, 0.04)	0.27	–0.09 (–0.17, 0.01)	0.04	0.003 (–0.08, 0.09)	0.94	–0.02 (0.11, 0.07)	0.69	–0.02 (–0.11, 0.08)	0.75	–0.05 (–0.14, 0.03)	0.22
Cortical content (SD)	0.04 (–0.05, 0.12)	0.37	0.04 (–0.05, 0.12)	0.43	0.04 (–0.05, 0.12)	0.43	–0.02 (–0.10, 0.06)	0.66	0.05 (–0.03, 0.14)	0.23	0.04 (–0.06, 0.13)	0.44	0.04 (–0.05, 0.13)	0.41	–0.02 (–0.10, 0.07)	0.72
Periosteal circum (SD)	0.14 (0.05, 0.22)	0.002	0.15 (0.06, 0.23)	< 0.001	0.14 (0.06, 0.23)	< 0.001	0.08 (0.01, 0.15)	0.02	0.15 (0.07, 0.24)	< 0.001	0.13 (0.04, 0.22)	0.004	0.13 (0.04, 0.22)	0.004	0.07 (–0.01, 0.14)	0.08
Endosteal circum (SD)	0.17 (0.08, 0.25)	< 0.001	0.19 (0.10, 0.27)	< 0.001	0.19 (0.10, 0.27)	< 0.001	0.15 (0.07, 0.24)	< 0.001	0.15 (0.06, 0.23)	0.001	0.14 (0.05, 0.23)	0.003	0.13 (0.04, 0.23)	0.004	0.10 (0.01, 0.19)	0.03

^a^ Model 1: Adjusted for child's gestational age at delivery, age at pQCT, and gender.

^b^ Model 2: Same as Model 1 and maternal age, height, weight, and parity at delivery.

^c^ Model 3: Same as Model 2 and child's pubertal stage at age 13.5 years.

^d^ Model 4: Same as Model 3 and child's height and weight at age 17.7 years.

**Table 4:** Associations Between Placental Characteristics and Childhood Bone DXA Measurements

	9.9 years			15.5 years			17.7 years		
Placental measure	WB BA (SD) B (95% CI)	WB BMC (SD) B (95% CI)	WB BMD (SD) B (95% CI)	WB BA (SD) B (95% CI)	WB BMC (SD) B (95% CI)	WB BMD (SD) B (95% CI)	WB BA (SD) B (95% CI)	WB BMC (SD) B (95% CI)	WB BMD (SD) B (95% CI)
Area (SD)	0.12^**^	0.10^*^	0.05	0.09^*^	0.07	0.03	0.10	0.08	0.04
	(0.03, 0.2)	(0.01, 0.18)	(–0.04, 0.14)	(0.01, 0.18)	(–0.01, 0.16)	(–0.06, 0.11)	(0.02, 0.18)^*^	(–0.001, 0.16)	(–0.04, 0.12)
Volume (SD)	0.14^**^	0.12^**^	0.08	0.12^**^	0.09	0.02	0.04	0.02	–0.02
	(0.05, 0.23)	(0.03, 0.22)	(–0.01, 0.17)	(0.04, 0.21)	(–0.001, 0.18)	(–0.07, 0.11)	(–0.01, 0.08)	(–0.03, 0.07)	(–0.09, 0.05)

All associations adjusted for child's gestational age at delivery, age at DXA, and gender.

WB = whole body (minus head); BA = bone area; BMC = bone mineral content; BMD = bone mineral density.

^*^ Significant at *p* < 0.05.

^**^ Significant at *p* < 0.01.

^***^Significant at *p* < 0.001.

### Placental size and offspring pQCT indices at age 17.7 years

Table 3 summarizes the relationships observed between placental measurements and offspring bone mass at age 17.7 years. Although the previously observed associations were attenuated, in the fully adjusted models including child's height and weight, positive relationships remained between placental size (area and volume) and EC and PC. The negative association between placental volume and cortical density remained, and that between placental area and cortical density was attenuated (*p* = 0.18). Relationships were similar in boys and girls. Again, inclusion of birth weight modestly attenuated the beta coefficients, but the overall pattern of associations remained similar (Supporting Table 5). In a further analysis, in which pQCT indices at age 17.7 years were conditioned on those at age 15.5 years, relationships were further attenuated (Supporting Table 6).

### Placental size and offspring DXA measurements of bone mass

At age 9.9 years, positive relationships were observed between each of placental area and volume, and offspring WB (minus head) BA and WB (minus head) BMC (Table 4), similar in boys and girls. No associations between placental measures and child WB (minus head) were seen. At age 15.5 years, there were similar positive associations between placental area or volume and DXA BA, but those with BMC were partially attenuated (*p* = 0.07 and 0.09, respectively). At age 17.7 years, the associations were attenuated further with only that between placental area and BA remaining statistically significant (Table 4), albeit with a beta coefficient of similar magnitude to that at earlier time points. Associations remained similar in Models 2 and 3, but inclusion of child's height and weight in Model 4 removed all relationships. Indeed, both placental area and placental volume, adjusted for gestational age at birth, gender, and age at the 9‐year visit, were weakly correlated with height at 9.9 years (*r*_partial_ for placental area = 0.08, *p* = 0.02; *r*_partial_ for placental volume = 0.13, *p* = 0.0001). Inclusion of birth weight in the regression analysis also attenuated associations with BA to below statistical significance (Supporting Table 7). Finally, in conditional analyses in which DXA indices at age 17.7 years were controlled for those at age 15.5 years (Supporting Table 8), and those at age 15.5 years were controlled for those at age 9.9 years (Supporting Table 9), in a similar pattern to those with pQCT, all associations were attenuated.

## Discussion

To our knowledge, this is the first study to investigate relationships between placental size and offspring bone indices using pQCT. We found that our previously documented positive associations between placental size and neonatal bone mass by DXA persisted into later childhood but appeared attenuated across puberty. However, placental area and volume were positively associated with offspring cortical area, PC, and EC at both age 15.5 and 17.7 years, even after adjustment for a range of covariates, including pubertal status and current body size. Conversely, placental size was negatively related to cortical density.

This was a large prospective cohort study, with detailed characterization of mothers and children, using two validated methods of bone mineral assessment; however, there are some limitations that should be considered when interpreting the results. First, the placentas were not measured immediately after collection, but were stored for several years in formaldehyde. The effect of this on placental size and shape is uncertain; nevertheless, because all placentas were stored identically, this is unlikely to have affected the relationship observed between placental size and offspring bone mass. Second, we have reported on a subset of the ALSPAC cohort, and thus there is the possibility that our participants may not be representative of the entire cohort, thereby reducing the generalizability of our findings. Again, because all analyses were within the present cohort, there is no reason to suppose that this would have influenced the associations observed. Third, only midtibial pQCT scans were performed so it is not possible to examine the placental influences on trabecular bone. Finally, although prospective, this was an observational study and so causality in any observed associations cannot be definitively established.

These results complement our previous findings from another UK mother–offspring cohort, the Southampton Women's Survey.^10^ Here, placental volume at 19 weeks' gestation, estimated from high‐resolution ultrasound measurements, was positively associated with whole‐body BA, BMC, and more weakly with BMD from DXA at birth. Similarly, in the current study we observed positive associations between placental area or volume and whole‐body DXA, BA, and BMC at 9 years, but much weaker associations with BMD. Interestingly, although the direction of associations was maintained, the magnitude of placenta‐bone associations was much attenuated by the age of 17.7 years, suggesting that pubertal transition might modify these relationships. However, in the present study, we also assessed children at ages 15.5 and 17.7 years using pQCT, which allowed detailed measurements of bone indices without the effect of overall size that confounds DXA measures. PC and EC were positively associated with placental size; nonetheless, we observed an inverse association between placental size and volumetric cortical BMD at the tibia, suggesting a disparity between influences on bone size and volumetric density, as had been observed with other aspects of intrauterine growth.^16^

The mechanisms that might underlie associations between placental size and offspring bone development are poorly characterized, but may comprise direct effects of the placenta on long‐term postnatal growth trajectories, shared determinants of placental size and bone indices, or mediation through factors such as age at pubertal onset. There is scant evidence to inform the first two hypotheses, although we have previously shown associations between patterns of intrauterine growth and postnatal skeletal development^5^, ^16^, ^17^; early growth, adult hip morphology,^6^, ^18^ and risk of hip fracture^7^, ^8^; and positive relationships between expression of placental calcium transporters and offspring BMC at birth.^19^

In relation to the third hypothesis, there may be two components to a potential maturational explanation for our findings. These children were assessed toward the end of the pubertal period, during which substantial linear growth had occurred. The concept of “cortical consolidation” describes the way in which mineralization may lag behind growth in bone size during modeling, with mineralization and volumetric density catching up with skeletal size by the time of peak bone mass.^20^ Indeed, late puberty is a time of rapid bone remodeling, with increased cortical porosity and active periosteal apposition—both characteristics that would be consistent with our findings.

One hypothesis, therefore, is that greater placental size leads to earlier onset of puberty, resulting in larger bones at age 15.5 years, but with cortical density lagging behind proportionate to bone size (with larger bones having lower cortical density compared with smaller bones). Clearly, such a mechanism was proposed in a recent study from the ALSPAC cohort,^21^ based on all children who underwent pQCT at ages 15.5 and 17.7 years, linking birth weight to bone outcomes. Here, relationships between birth weight and pQCT measures were somewhat attenuated by adjustment for puberty, and those with cortical density were not apparent at age 17.7 years. We found that associations between placental size and pQCT measures at age 15.5 years were not appreciably changed by adjustment for puberty; however, relationships between placental size and pQCT measures at age 17.7 years, although robust for PC and EC, were much weaker for cortical density, consistent with a maturational etiology and further supported by the conditional models, showing that the strongest placental associations were with the earlier time points of follow‐up. Conversely, whereas increasing pubertal stage at age 13.5 years was associated with larger bones by pQCT, there was no evidence of placental size having been greater in children who were at a later stage of puberty at age 13.5 years. Additionally, increasing pubertal stage at age 13.5 years was associated with increasing rather than decreasing cortical density. It must be noted however that the 2‐year interval between pubertal staging and pQCT measures somewhat limits the inferences that can be made. Furthermore, the correlation between birth weight and placental area was 0.4, suggesting much scope for relationships between placental size and outcomes independent of birth weight, consistent with previous documentation of the role of placental size versus function.^22^, ^23^ Inclusion of birth weight in the base model removed associations between placental size and DXA BA, most likely due to the strong association between birth weight and overall size, thus potentially on the causal pathway. In contrast, associations between placental size and pQCT measures of PC, EC, and cortical density, although attenuated, remained similar to those without the inclusion of birth weight, suggesting relationships over and above those mediated through size at birth. Consistent with these findings, although placental size was weakly correlated with height in childhood, and whereas the DXA associations were removed by addition of height in the models, those with the pQCT indices remained statistically significant, further supporting the notion that the placenta pQCT relationships were not purely mediated via linear growth.

Second, it is notable from pQCT studies that bone size, for example, PC, tends to be inversely related to cortical density.^24^ The bending strength of a bone is proportional to the fourth power of the radius^25^ and thus greater diameter bones require lower cortical density to achieve the same strength as narrower bones.^26^ Because the skeleton adapts its structure to the prevailing loads imposed on it, and cortical density encompasses cortical porosity as well as tissue mineralization, this then provides a second possible mechanism. Certainly, when both PC and cortical density were regressed simultaneously on placental volume or area, the predominant association was with PC, suggesting that the primary effect is on bone size—an observation that could support either of these two maturational hypotheses.

Both of these potential explanations would be compatible with the observed increased incidence of childhood fractures during the transition into puberty, where increase in bone size appears to outstrip mineralization^27^; reassuringly, however, the relative catch‐up in mineralization by young adulthood^20^ suggests that by the time peak bone mass has been achieved, a larger placenta is likely to be associated with greater adult bone strength.

In conclusion, we have shown that previously observed associations between placental size and offspring bone size persist into late childhood, and that placental size is differentially related to bone size and volumetric density over the pubertal period. Whereas these results further our understanding of relationships between early development and postnatal skeletal growth, the magnitudes of association are modest by late adolescence and the elucidation of underlying mechanisms must remain a subject for further investigation.

## Disclosures

All authors state that they have no conflicts of interest.

## Supporting information

Supporting Information.

Supporting Information.
