# Supplementary material for: Placental Size Is Associated Differentially With Postnatal Bone Size and Density
Source: J Bone Miner Res. 2016 Apr 22;31(10):1855–64. doi: 10.1002/jbmr.2840 (PMC5010780; doi:10.1002/jbmr.2840)
Supplement: Supplementary file 2 — Supporting Information. [file JBMR-31-1855-s002.docx]

**Supporting Fig. 1.** Participant numbers

**
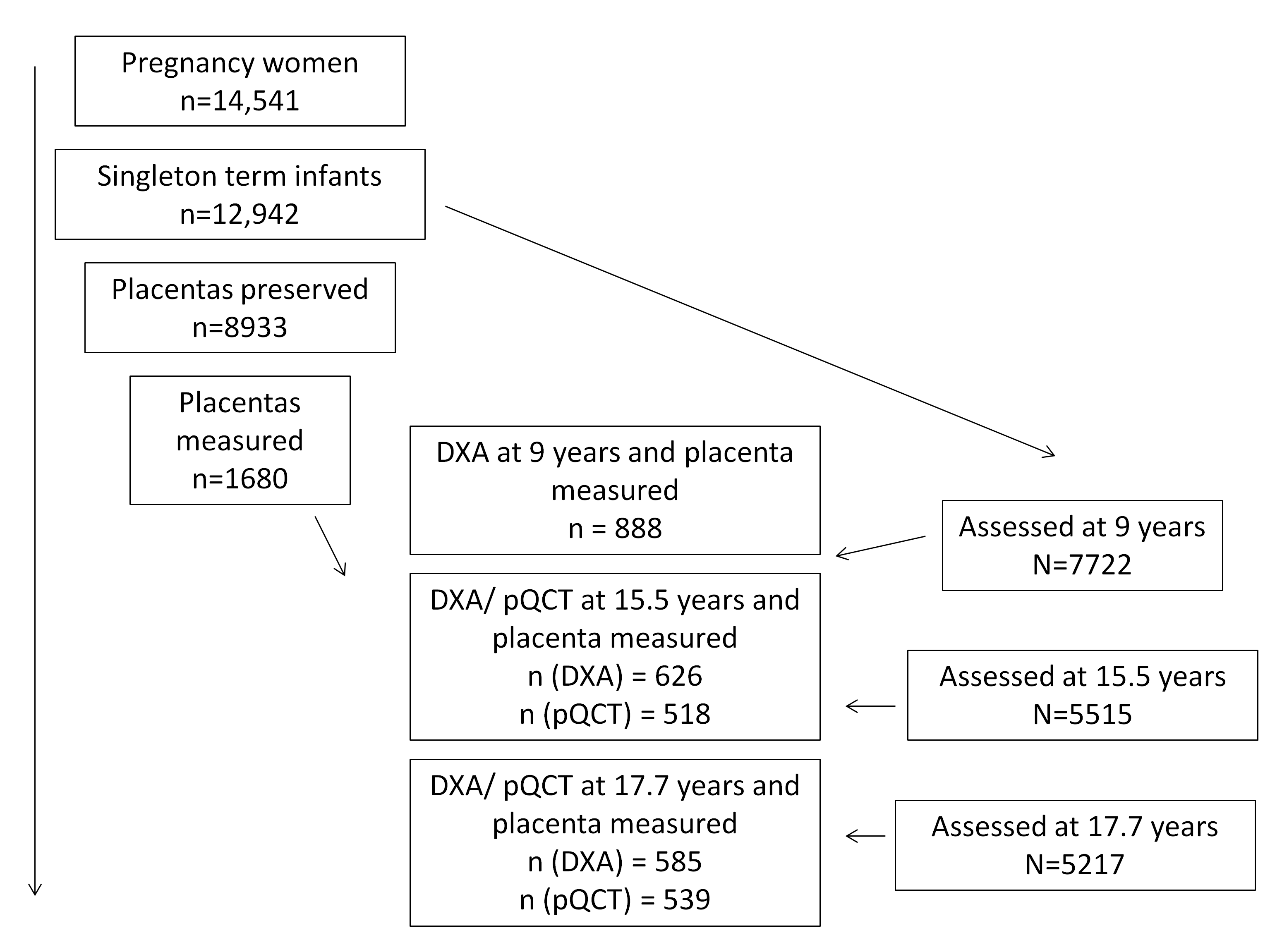
**

**Supporting Table 1.** DXA indices at 9.9, 15.5 and 17.7 years

|  | **Boys** | | |  | **Girls** | | |  |
| --- | --- | --- | --- | --- | --- | --- | --- | --- |
|  | n | Mean | SD |  | n | Mean | SD | p diff. |
| **DXA at 9.9 years** |  |  |  |  |  |  |  |  |
| Total area less head (cm^2^) | 213 | 1141.5 | 156.3 |  | 268 | 1128.1 | 163.8 | 0.4 |
| Total BMC less head (g) | 213 | 900.5 | 172.6 |  | 268 | 880.6 | 181.6 | 0.2 |
| Total BMD less head (g/cm^2^) | 213 | 0.8 | 0.05 |  | 268 | 0.8 | 0.05 | 0.06 |
|  |  |  |  |  |  |  |  |  |
| **DXA at 15.5 years** |  |  |  |  |  |  |  |  |
| Total area less head (cm^2^) | 230 | 2103.7 | 276.2 |  | 288 | 1918.1 | 229.4 | <0.001 |
| Total BMC less head (g) | 230 | 2261.9 | 478.5 |  | 288 | 1945.8 | 353.1 | <0.001 |
| Total BMD less head (g/cm^2^) | 230 | 1.1 | 0.1 |  | 288 | 1.0 | 0.1 | <0.001 |
|  |  |  |  |  |  |  |  |  |
| **DXA at 17.7 years** |  |  |  |  |  |  |  |  |
| Total area less head (cm^2^) | 242 | 2251.3 | 253.8 |  | 343 | 1956.9 | 251.6 | <0.001 |
| Total BMC less head (g) | 242 | 2603.9 | 482.4 |  | 343 | 2052.3 | 381.0 | <0.001 |
| Total BMD less head (g/cm^2^) | 242 | 1.1 | 0.1 |  | 343 | 1.0 | 0.1 | <0.001 |

WB = Whole body minus head; BA= Bone Area; BMC = Bone Mineral Content; BMD = Bone Mineral Density

**Supporting Table 2.** Associations between placental characteristics and pQCT measurements at 15.5 years (adjusted for birthweight)

|  |  | | |  | | |
| --- | --- | --- | --- | --- | --- | --- |
| **Placental measure** | **Cortical area (SD)** | **Cortical density (SD)** | **Cortical thickness (SD)** | **Cortical content (SD)** | **Periosteal circumference (SD)** | **Endosteal circumference (SD)** |
|  | B (95%CI) | B (95%CI) | B (95%CI) | B (95%CI) | B (95%CI) | B (95%CI) |
| **Area (SD)** | 0.04  (-0.06, 0.13) | -0.12*  (-0.22,-0.03) | -0.07  (-0.17, 0.03) | 0.01  (-0.09. 0.10) | 0.12  (0.03, 0.22)** | 0.18  (0.09, 0.27)*** |
| **Volume (SD)** | 0.07  (-0.03, 0.17) | -0.10  (-0.20, 0.01) | -0.04  (-0.14, 0.07) | 0.04  (-0.06, 0.15 | 0.14  (0.04, 0.25)** | 0.16  (0.06, 0.27)** |

WB = Whole body minus head; BA= Bone Area; BMC = Bone Mineral Content; BMD = Bone Mineral Density; All associations adjusted for child’s gestational age at delivery, age at DXA, sex and birthweight; *p<0.05; **p<0.01; ***p<0.001

**Supporting Table 3A.** Association between pubertal stage at 13.5 years and placental measurements in boys (complete case analysis)

|  | **Placental measurement** | | | |
| --- | --- | --- | --- | --- |
| **Pubertal stage** (n=143) | **Area (cm^2^)** | | **Volume (cm^3^)** | |
|  | mean | SD | mean | SD |
| 1 (n=17  2 (n=32) | 272.6  296.0 | 43.9  59.8 | 764.7  855.5 | 163.6  218.2 |
| 3 (n=42) | 281.4 | 59.0 | 757.1 | 176.8 |
| 4 (n=41) | 279.4 | 55.0 | 767.7 | 172.2 |
| 5 (n=11) | 283.1 | 36.6 | 758.4 | 127.9 |
|  |  |  |  |  |
| **Test for linear trend (p value)** | 0.8 | | 0.2 | |

**Supporting Table 3B.** Association between pubertal stage at 13.5 years and placental measurements in girls (complete case analysis)

|  | **Placental measurement** | | | |
| --- | --- | --- | --- | --- |
| **Pubertal stage** (n=212) | Area (cm^2^) | | Volume (cm^3^) | |
|  | mean | SD | mean | SD |
| 1 (n=11)  2 (n=21)  3 (n=50) | 307.8  272.9  277.3 | 36.7  47.4  46.1 | 799.1  771.3  813.8 | 157.8  195.3  132.7 |
| 4 (n=87) | 285.4 | 57.1 | 782.2 | 168.1 |
| 5 (n=43) | 289.3 | 66.6 | 807.4 | 226.3 |
|  |  |  |  |  |
| **Test for linear trend (p value)** | 0.8 | | 0.9 | |

**Supporting Table 4A.** Association between pubertal stage at 13.5 years and pQCT measurements at 15.5 years in boys (complete case analysis)

|  | **pQCT measurement (mean (SD))** | | | | | |
| --- | --- | --- | --- | --- | --- | --- |
| **Pubertal stage** (n=143) | Cortical area  (cm^2^) | Cortical BMD (mg/cm^2^) | Cortical thickness  (mm) | Cortical content  (mg) | Periosteal circumference  (mm) | Endosteal circumference  (mm) |
|  |  |  |  |  |  |  |
| 1 (n=17) | 305.3  (40.6) | 1055.9  (42.3) | 5.4  (0.4) | 322.2  (44.1) | 73.7  (5.5) | 40.0  (4.7) |
| 2 (n=32) | 331.3  (44.8) | 1048.4  (28.9) | 5.6  (0.6) | 347.4  (48.8) | 76.7  (6.2) | 41.2  (7.3) |
| 3 (n=42) | 328.4  (42.0) | 1076.1  (35.3) | 5.6  (0.6) | 353.5  (47.5) | 76.3  (4.9) | 41.1  (5.0) |
| 4 (n=41) | 339.8  (48.2) | 1098.2  (28.1) | 5.8  (0.7) | 373.3  (53.9) | 76.6  (5.0) | 40.0  (4.6) |
| 5 (n=11) | 345.9  (57.9) | 1104.8  (24.6) | 6.0  (0.8) | 381.5  (59.3) | 76.4  (5.8) | 38.6  (5.8) |
|  |  |  |  |  |  |  |
| **Linear test for trend (p value)** | **0.01** | **<0.001** | **0.003** | **<0.001** | 0.2 | 0.4 |

**Supporting Table 4B.** Association between pubertal stage at 13.5 years and pQCT measurements at 15.5 years in girls (complete case analysis)

|  | **pQCT measurement (mean (SD))** | | | | | |
| --- | --- | --- | --- | --- | --- | --- |
| **Pubertal stage** (n=212) | Cortical area  (cm^2^) | Cortical BMD (mg/cm^2^) | Cortical thickness  (mm) | Cortical content  (mg) | Periosteal circumference  (mm) | Endosteal circumference  (mm) |
|  |  |  |  |  |  |  |
| 1 (n=11) | 265.4  (34.1) | 1093.4  (24.5) | 5.0  (0.6) | 290.2  (38.0) | 69.1  (3.2) | 37.8  (3.9) |
| 2 (n=21) | 261.9  (36.4) | 1115.6  (25.7) | 5.0  (0.6) | 292.2  (40.4) | 67.8  (4.4) | 36.1  (4.6) |
| 3 (n=50) | 276.3  (34.8) | 1128.2  (18.8) | 5.2  (0.6) | 311.8  (40.0) | 69.2  (4.0) | 36.2  (4.2) |
| 4 (n=87) | 283.3  (38.5) | 1126.8  (23.4) | 5.3  (0.6) | 319.2  (43.4) | 69.8  (5.0) | 36.2  (5.6) |
| 5 (n=43) | 282.2  (30.9) | 1135.5  (20.0) | 5.4  (0.5) | 320.0  (33.2) | 69.5  (4.1) | 35.8  (4.5) |
|  |  |  |  |  |  |  |
| **Linear test for trend (p value)** | **0.01** | **<0.001** | **0.005** | **0.001** | 0.2 | 0.4 |

**Supporting** **Table 5.** Associations between placental characteristics and pQCT measurements at 17.7 years (adjusted for birthweight)

| **Placental measure** | **Cortical area (SD)** | **Cortical density (SD)** | **Cortical thickness (SD)** | **Cortical content (SD)** | **Periosteal circumference (SD)** | **Endosteal circumference (SD)** |
| --- | --- | --- | --- | --- | --- | --- |
|  | B (95%CI) | B (95%CI) | B (95%CI) | B (95%CI) | B (95%CI) | B (95%CI) |
| **Area (SD)** | -0.01  (-0.10, 0.09) | -0.05  (-0.14, 0.04) | -0.07  (-0.16, 0.02) | -0.01  (-0.10, 0.08) | 0.07  (-0.02, 0.16) | 0.13  (0.04, 0.22)** |
| **Volume (SD)** | 0.01  (-0.09, 0.10) | -0.11  (-0.21, -0.01)* | -0.03  (-0.13, 0.07) | -0.01  (-0.11, 0.09) | 0.08  (-0.02, 0.17) | 0.10  (<0.001, 0.20) |

WB = Whole body minus head; BA= Bone Area; BMC = Bone Mineral Content; BMD = Bone Mineral Density; All associations adjusted for child’s gestational age at delivery, age at DXA, sex and birthweight; *p<0.05; **p<0.01; ***p<0.001

**Supporting Table 6.** Associations between placental characteristics and childhood pQCT measurements at 17.7 yrs (adjusted for pQCT at 15.5 yrs)

| pQCT at 17.7 years | Placental measurement | | | | | | | | | | | | | | | |
| --- | --- | --- | --- | --- | --- | --- | --- | --- | --- | --- | --- | --- | --- | --- | --- | --- |
|  | **Area (SD)** | | | | | | | | **Volume (SD)** | | | | | | | |
|  | B^1^  (95% CI) | p | B^2^  (95% CI) | p | B^3^  (95% CI) | p | B^4^ | p | B^1^  (95% CI) | p | B^2^  (95% CI) | p | B^3^  (95% CI) | p | B^4^ | p |
| Cortical area (SD) | -0.03  (-0.08, 0.02) | 0.28 | -0.02  (-0.08, 0.03) | 0.39 | -0.03  (-0.08, 0.03) | 0.33 | -0.03  (-0.08, 0.02) | 0.29 | 0.001  (-0.05, 0.06) | 0.97 | 0.01  (-0.05, 0.07) | 0.66 | 0.001  (-0.06, 0.06) | 0.96 | <0.001  (-0.06, 0.06) | 0.99 |
| Cortical BMD (SD) | -0.003  (-0.08, 0.08) | 0.94 | 0.002  (-0.08, 0.08) | 0.97 | 0.003  (-0.08, 0.08) | 0.94 | 0.02  (-0.06, 0.10) | 0.69 | -0.01  (-0.09, 0.07) | 0.79 | -0.001  (-0.09, 0.09) | 0.99 | -0.002  (-0.10, 0.09) | 0.97 | 0.01  (-0.08, 0.10) | 0.80 |
| Cortical thickness (SD) | -0.01  (-0.05, 0.03) | 0.64 | -0.01  (-0.05, 0.04) | 0.80 | -0.01  (-0.05, 0.04) | 0.82 | -0.01  (-0.05, 0.03) | 0.53 | 0.02  (-0.02, 0.06) | 0.31 | 0.02  (-0.02, 0.07) | 0.34 | 0.02  (-0.03, 0.06) | 0.44 | 0.01  (-0.03, 0.06) | 0.64 |
| Cortical content (SD) | -0.01  (-0.07, 0.04) | 0.64 | -0.01  (-0.06, 0.05) | 0.87 | -0.01  (0.06, 0.05) | 0.81 | -0.01  (-0.06, 0.05) | 0.79 | 0.02  (-0.04, 0.07) | 0.58 | 0.03  (-0.03, 0.09) | 0.31 | 0.02  (-0.04, 0.08) | 0.57 | 0.02  (-0.04, 0.08) | 0.56 |
| Periosteal circum (SD) | -0.01  (-0.06, 0.04) | 0.73 | -0.002  (-0.05, 0.05) | 0.92 | -0.004  (-0.05, 0.04) | 0.87 | -0.01  (-0.05, 0.04) | 0.83 | 0.002  (-0.05, 0.05) | 0.94 | 0.01  (-0.04, 0.06) | 0.69 | 0.001  (-0.05, 0.05) | 0.96 | 0.001  (-0.05, 0.05) | 0.96 |
| Endosteal circum (SD) | 0.01  (-0.04, 0.06) | 0.06 | 0.01  (-0.04, 0.06) | 0.74 | 0.01  (-0.04, 0.06) | 0.72 | 0.01  (-0.05, 0.06) | 0.85 | -0.01  (-0.06, 0.04) | 0.69 | -0.01  (-0.06, 0.05) | 0.82 | -0.01  (-0.06, 0.05) | 0.76 | -0.01  (-0.07, 0.04) | 0.66 |

^1^Model 1: Adjusted for child’s gestational age at delivery, age at pQCT and sex; ^2^ Model 2: As model 1 and maternal age at delivery, height, weight and parity

^3^Model 3: As model 2 and child’s pubertal stage at 13.5 years; ^4^Model 4: As model 3 and child’s height and weight at 17.7 years

**Supporting Table 7.** Associations between placental characteristics and childhood DXA measurements (adjusted for birthweight)

|  | **9 years** | | | **15.5 years** | | |  | **17.7 yrs** |  |
| --- | --- | --- | --- | --- | --- | --- | --- | --- | --- |
| **Placental measure** | **WB BA (SD)** | **WB BMC (SD)** | **WB BMD (SD)** | **WB BA (SD)** | **WB BMC (SD)** | **WB BMD (SD)** | **WB BA**  **(SD)** | **WB BMC**  **(SD)** | **WB BMD**  **(SD)** |
|  | B (95%CI) | B (95%CI) | B (95%CI) | B (95%CI) | B (95%CI) | B (95%CI) | B (95%CI) | B (95%CI) | B (95%CI) |
| **Area (SD)** | 0.003  (-0.06, 0.07) | 0.003  (-0.07, 0.07) | -0.001  (-0.07, 0.07) | -0.02  (-0.10,0.07) | -0.02  (-0.10, 0.07) | -0.03  (-0.11, 0.06) | <0.001  (-0.08, 0.08) | -0.003  (-0.09, 0.08) | -0.01  (-0.10, 0.08) |
| **Volume (SD)** | 0.04  (-0.03, 0.12) | 0.05  (-0.03, 0.12) | 0.03  (-0.05, 0.11) | 0.02  (-0.07,0.11) | 0.02  (-0.08, 0.11) | -0.01  (-0.10, 0.08) | 0.06  (-0.03, 0.15) | 0.04  (-0.05, 0.13) | 0.003  (-0.09, 0.10) |

WB = Whole body minus head; BA= Bone Area; BMC = Bone Mineral Content; BMD = Bone Mineral Density; All associations adjusted for child’s gestational age at delivery, age at DXA, sex and birthweight; all p>0.05

**Supporting Table 8.** Associations between placental characteristics and childhood DXA measurements at 17.7 yrs (adjusted for DXA at 15.5 yrs)

| DXA at 17.7 years | Placental measurement | | | | | | | | | | | | | | | |
| --- | --- | --- | --- | --- | --- | --- | --- | --- | --- | --- | --- | --- | --- | --- | --- | --- |
|  | **Area (SD)** | | | | | | | | **Volume (SD)** | | | | | | | |
|  | B^1^  (95% CI) | p | B^2^  (95% CI) | p | B^3^  (95% CI) | p | B^4^ | p | B^1^  (95% CI) | p | B^2^  (95% CI) | p | B^3^  (95% CI) | p | B^4^ | p |
| WB BA (SD) | -0.01  (-0.06, 0.04) | 0.66 | -0.003  (-0.05, 0.04) | 0.90 | -0.10  (-0.06, 0.04) | 0.72 | -0.02  (-0.05, 0.02) | 0.38 | 0.04  (-0.01, 0.08) | 0.16 | 0.03  (-0.02, 0.08) | 0.25 | 0.02  (-0.03, 0.07) | 0.49 | 0.01  (-0.03, 0.05) | 0.50 |
| WB BMC  (SD) | -0.02  (-0.06, 0.02) | 0.37 | -0.01  (-0.05, 0.03) | 0.56 | -0.02  (-0.06, 0.02) | 0.38 | -0.03  (-0.06, 0.003) | 0.07 | 0.02  (-0.02, 0.06) | 0.32 | 0.02  (-0.03, 0.06) | 0.46 | 0.004  (-0.04, 0.05) | 0.85 | -0.01  (-0.04, 0.03) | 0.70 |
| WB BMD  (SD) | -0.02  (-0.06, 0.03) | 0.46 | -0.01  (-0.05, 0.03) | 0.65 | -0.01  (-0.05, 0.03) | 0.54 | -0.03  (-0.07, 0.01) | 0.15 | 0.01  (-0.03, 0.06) | 0.06 | 0.01  (-0.03, 0.06) | 0.59 | 0.002  (-0.04, 0.04) | 0.91 | -0.01  (-0.05, 0.03) | 0.55 |

WB = Whole body minus head; BA= Bone Area; BMC = Bone Mineral Content; BMD = Bone Mineral Density

^1^Model 1: Adjusted for child’s gestational age at delivery, age at pQCT and sex; ^2^ Model 2: As model 1 and maternal age at delivery, height, weight and parity

^3^Model 3: As model 2 and child’s pubertal stage at 13.5 years; ^4^Model 4: As model 3 and child’s height and weight at 17.7 years

**Supporting Table 9.** Associations between placental characteristics and childhood DXA measurements at 15.5 yrs (adjusted for DXA at 9 yrs)

| DXA at 15.5 years | Placental measurement | | | | | | | | | | | | | | | |
| --- | --- | --- | --- | --- | --- | --- | --- | --- | --- | --- | --- | --- | --- | --- | --- | --- |
|  | **Area (SD)** | | | | | | | | **Volume (SD)** | | | | | | | |
|  | B^1^  (95% CI) | p | B^2^  (95% CI) | p | B^3^  (95% CI) | p | B^4^ | p | B^1^  (95% CI) | p | B^2^  (95% CI) | p | B^3^  (95% CI) | p | B^4^ | p |
| WB BA (SD) | -0.003  (-0.05, 0.04) | 0.89 | 0.002  (-0.05, 0.05) | 0.93 | 0.002  (-0.05, 0.05) | 0.94 | -0.02  (-0.05, 0.02) | 0.29 | 0.01  (-0.04, 0.06) | 0.68 | 0.01  (-0.04, 0.06) | 0.78 | 0.01  (-0.06, 0.07) | 0.85 | 0.01  (-0.04, 0.06) | 0.73 |
| WB BMC  (SD) | -0.01  (-0.05, 0.04) | 0.84 | -0.01  (-0.05, 0.04) | 0.77 | -0.004  (-0.05, 0.04) | 0.87 | -0.03  (-0.07, 0.002) | 0.06 | -0.01  (-0.06, 0.03) | 0.58 | -0.02  (-0.07, 0.03) | 0.41 | -0.03  (-0.09, 0.02) | 0.25 | -0.02  (-0.06, 0.03) | 0.51 |
| WB BMD  (SD) | 0.01  (-0.04, 0.06) | 0.75 | -0.002  (-0.05, 0.05) | 0.94 | 0.004  (-0.04, 0.05) | 0.88 | -0.04  (-0.08, 0.005) | 0.09 | -0.02  (-0.07, 0.04) | 0.53 | -0.03  (-0.08, 0.02) | 0.25 | -0.04  (-0.10, 0.01) | 0.14 | -0.02  (-0.07, 0.03) | 0.34 |

WB = Whole body minus head; BA= Bone Area; BMC = Bone Mineral Content; BMD = Bone Mineral Density

^1^Model 1: Adjusted for child’s gestational age at delivery, age at pQCT and sex; ^2^ Model 2: As model 1 and maternal age at delivery, height, weight and parity

^3^Model 3: As model 2 and child’s pubertal stage at 13.5 years; ^4^Model 4: As model 3 and child’s height and weight at 15.5 years
