# Supplementary material for: Placental Size Is Associated Differentially With Postnatal Bone Size and Density
Source: J Bone Miner Res. 2016 Apr 22;31(10):1855–64. doi: 10.1002/jbmr.2840 (PMC5010780; doi:10.1002/jbmr.2840)
Supplement: Supplementary file 1 — Supporting Information. [file JBMR-31-1855-s001.docx]

Juliet Compston

Editor-in-Chief

Journal of Bone and Mineral Research

12^th^ February 2016

**Re: Placental size is associated differentially with postnatal bone size and density**

Dear Juliet,

Thank you very much for considering our paper for publication in the Journal of Bone and Mineral Research. We also thank the reviewers for their helpful comments, and have extensively modified the manuscript in the light of these suggestions, with the detailed responses itemised below:

**Reviewer: 1**

**Comments to the Author**

**The article presents cross-sectional associations between measures of placental size and bone outcomes at different stages of childhood and adolescence in over 500 children from the ALSPAC cohort.**

**1. A main advantage of the ALSPAC cohort is the collection of longitudinal data, yet in these analyses associations have been limited to each separate time point. The main finding relates to bone size and placental area/ volume. Was the relationship between placental size and change in bone over time considered? For DXA I think this could have consisted three time points (9, 15 and 17 years) and for pQCT, two (15 and 17 years).**

We thank the reviewer for this suggestion. We had considered in some detail what to include in the paper and were cognisant of space constraints, so elected to focus on the absolute measures at sequential follow-ups in this prospective cohort. However we agree that it would be helpful to incorporate some assessment of change and have added a brief further analysis using multiple regression to document any additional association between placental size and offspring bone at later timepoints when conditioned on bone at early timepoints. We have added a description to the methods, and results and discussion sections.

Methods: page 7, para 2 to page 8, para 1:

*“In order to assess the contribution of placental size to change in bone outcomes, we used a conditional multiple regression analysis, in which the 4 models were used as before, but for each pQCT or DXA measure, we additionally included the corresponding measure at the previous time point. This effectively yields the association between placental size and the bone measure at a later time point, over and above that explained by the association between placental size and the bone measure at the earlier time point. We undertook separate analyses to assess whether associations might be mediated via birthweight and by including birthweight in model 1. Placental measurements and sex interactions were examined, however these provided little evidence of sex differences and we therefore analysed boys and girls together. In line with convention, DXA-derived whole body bone variables were analysed minus head. To enable comparison of effect sizes across relationships, all predictor and outcome variables were standardised to z-scores with a mean of 0 and an SD of 1. Regression coefficients are therefore representative of SD change in outcome per unit SD change in predictor, and may be interpreted as partial correlation coefficients. Analysis was performed using SPSS Version 21 (IBM UK Ltd, Portsmouth, UK).”*

Results: page 10, para 2 to page 11, para 1:

*“Placental size and offspring pQCT indices at age 17.7 years*

*Table 3 summarises the relationships observed between placental measurements and offspring bone mass at age 17.7 years. Although the previously observed associations were attenuated, in the fully adjusted models, positive relationships remained between placental size (area and volume) and endosteal circumference and periosteal circumference. The negative association between placental volume and cortical density remained, and that between placental area and cortical density was partially attenuated (p=0.18). Relationships were similar in boys and girls. Again, inclusion of birthweight modestly attenuated the beta coefficients, but the overall pattern of associations remained similar (Online Supplementary Table 5). In a further analysis, in which pQCT indices at age 17.7 years were conditioned on those at 15.5 years, relationships were further attenuated (Online Supplementary Table 6).*

*Placental size and offspring DXA measurements of bone mass*

*At age 9.9 years, positive relationships were observed between each of placental area and volume, and offspring WB (minus head) BA and WB (minus head) BMC (Table 4), similar in boys and girls. No associations between placental measures and child BMD (minus head) were seen. At 15.5 years, similar positive associations between placental area or volume and DXA BA, but those with BMC were partially attenuated (p=0.07 and 0.09 respectively). At 17.7 years, the associations were attenuated further with only that between placental area and BA remaining statistically significant (Table 4), albeit with a beta coefficient of similar magnitude to that at earlier timepoints. Associations remained similar in models 2 and 3, but inclusion of child’s height and weight in model 4 removed all relationships. Inclusion of birthweight in the regression analysis also attenuated associations with bone area to below statistical significance (Online Supplementary Table 7). Finally, in conditional analyses in which DXA indices at 17.7 years were controlled for those at 15.5 years (Online Supplementary Table 8), and those at 15.5 years were controlled for those that 9.9 years (Online Supplementary Table 9), in a similar pattern to those with pQCT, all associations were attenuated.”*

Discussion: page 13, para 2 to page 14, para 1:

*“One hypothesis therefore, is that greater placental size leads to earlier onset of puberty, resulting in larger bones at 15.5 years, but with cortical density lagging behind proportionate to bone size (with larger bones at having lower cortical density compared with smaller bones). Indeed, such a mechanism was proposed in a recent study from ALSPAC cohort (**21**), based on all children who underwent pQCT at 15.5 and 17.7 years, linking birthweight to bone outcomes. Here, relationships between birthweight and pQCT measures were somewhat attenuated by adjustment for puberty, and those with cortical density were not apparent at 17.7 years. We found that associations between placental size and pQCT measures at 15.5 years were not appreciably changed by adjustment for puberty; however relationships between placental size and pQCT measures at 17.7 years, whilst robust for periosteal and endosteal circumference, were much weaker for cortical density, consistent with a maturational etiology, and further supported by the conditional models, demonstrating that the strongest placental associations with the earlier time points of follow-up. Conversely, whilst increasing pubertal stage at 13.5 years was associated with larger bones by pQCT, there was no evidence of placental size having been greater in children who were at a later stage of puberty at 13.5 years. Additionally, increasing pubertal stage at 13.5 years was associated with increasing, rather than decreasing cortical density. It must be noted though that the 2 year interval between pubertal staging and pQCT measures somewhat limits the inferences that can be made. Furthermore, the correlation between birthweight and placental area was 0.4, suggesting much scope for relationships between placental size and outcomes independent of birthweight, consistent with previous documentation of the role of placental size versus function (**22**,**23**). Inclusion of birthweight in the base model removed associations between placental size and DXA bone area, most likely due to the strong association between birthweight and overall size, thus potentially on the causal pathway. In contrast, associations between placental size and pQCT measures of periosteal and endosteal circumference, and cortical density, although attenuated, remained similar to those without the inclusion of birthweight, suggesting relationships over and above those mediated through size at birth. Consistent with these findings, although placental size was weakly correlated with height in childhood, and whilst the DXA associations were removed by addition of height in the models, those with the pQCT indices remained statistically significant, further supporting the notion that the placenta pQCT relationships were not purely mediated via linear growth.”*

**2. Why were only the age 9 and 15 time points included for DXA? Can further justification for this selection be given? Also, it wasn't until pages 6 and 7 that the age 17.7 time point was mentioned.**

We apologise that this was not clearly articulated. We wished the main focus of the paper to be the pQCT measurements at 15 and 17 years as these added novel volumetric and structural information. The inclusion of DXA was really to demonstrate consistency with our previous publication in the Southampton Women’s Survey. However, we agree that it is reasonable to add in the DXA outcomes at 17 years and have done so in the supplementary information, together with text added to the results section. Finally we have now mentioned the 17 year timepoint earlier in the methods.

Methods: page 5, para 3 to page 6, para 1:

*“Skeletal assessment at 9.9, 15.5 and 17.7 years*

*Children were invited to attend the Focus@9 research clinic at age 9 years, in which whole body DXA assessment of bone mass by DXA (Lunar Prodigy, GE Corporation, Wisconsin, USA) was undertaken. At approximately 15.5 years of age, all children within the ALSPAC cohort were invited to attend a research clinic as part of a study investigating the effects of physical activity on cortical bone (**15**). Height (to the nearest 0.1cm) and weight (to the nearest 50g) were measured using a Harpenden Stadiometer (Holtain Ltd, Crymlych Wales) and Tanita Body Fat Analyser (Tanita UK Ltd, Uxbridge, UK) respectively. Measurements were then made of whole body bone area (BA), bone mineral content (BMC) and areal bone mineral density (BMD) using a DXA scanner with specific paediatric software (Lunar Prodigy, GE Corporation, Wisconsin, USA). The children also underwent pQCT assessment of their mid (50% from the distal endplate) right tibia using a Stratec XCT2000L instrument (Stratec, Pforzheim, Germany). Cortical BMD (BMD_C_) and cortical BMC (BMC_C_) were obtained. Periosteal circumference (PC), endosteal circumference (EC) and cortical thickness (CT) were derived using a circular ring model. Cortical bone was defined using a threshold above 650 mg/cm^3^, as previously described (**15**). The within subject coefficients of variation (CV) for pQCT measurements are displayed in parentheses: tibial length (4.04%), BMC_C_ (2.71%), BMD_C_ (1.29%), PC (1.58%), EC (4.03%). All scans were reviewed and those with artefact were excluded from analysis. Whole body DXA and tibial pQCT assessments were repeated in the cohort at approximately 17.7 years of age using identical methods.”*

Results: page 10, para 3 to page 11, para 1:

*“Placental size and offspring DXA measurements of bone mass*

*At age 9.9 years, positive relationships were observed between each of placental area and volume, and offspring WB (minus head) BA and WB (minus head) BMC (Table 4), similar in boys and girls. No associations between placental measures and child BMD (minus head) were seen. At 15.5 years, similar positive associations between placental area or volume and DXA BA, but those with BMC were partially attenuated (p=0.07 and 0.09 respectively). At 17.7 years, the associations were attenuated further with only that between placental area and BA remaining statistically significant (Table 4), albeit with a beta coefficient of similar magnitude to that at earlier timepoints. Associations remained similar in models 2 and 3, but inclusion of child’s height and weight in model 4 removed all relationships. Inclusion of birthweight in the regression analysis also attenuated associations with bone area to below statistical significance (Online Supplementary Table 7). Finally, in conditional analyses in which DXA indices at 17.7 years were controlled for those at 15.5 years (Online Supplementary Table 8), and those at 15.5 years were controlled for those that 9.9 years (Online Supplementary Table 9), in a similar pattern to those with pQCT, all associations were attenuated..”*

**3. Were there correlations between placental size and height? A larger cross-section is advantageous in a bone of the same length, therefore determining whether the link between the placenta and bone size is through height alone (through allometric scaling, Leonard JBMR 2004), or because it determines a wider bone would be interesting and helpful for interpretation of the results.**

This is a very good point, and we have added this information to the results. We did adjust for height in model 4, and the associations were not appreciably changed for pQCT outcomes, although were substantially attenuated for the DXA indices, as might be expected from their greater size-dependence, suggesting that the pQCT associations were not through height alone. We have clarified this point in the discussion.

Results: page 9, para 1:

*“Placental size and offspring pQCT indices at age 15.5 years*

*Table 2 summarises the relationships observed between placental measurements and offspring bone mass. We observed strong positive relationships between placental area and child’s cortical area, periosteal circumference and endosteal circumference at age 15.5 years, which remained robust after adjusting for gestational age, age at pQCT and sex (all p<0.05). Conversely there was a negative association between placental area and cortical BMD [β= -0.1 (95%CI: -0.19, -0.02); p=0.01]. These relationships remained, but were attenuated, after additional adjustments for maternal age at delivery, maternal parity, height and weight, and also after inclusion of child’s pubertal stage at 13.5 years, (except for placental area and cortical area, p=0.06; Table 2, Figure 2). Placental area, adjusted for gestational age, sex and age at pQCT, was weakly correlated with height at 15.5 years (r_partial_=0.07, p=0.048), as was placental volume (r_partial_: 0.1, p=0.03), but adjustment for child’s height and weight at 15.5 years did not materially alter the associations observed. Inclusion of birthweight modestly attenuated the beta coefficients, but the overall pattern of associations remained similar (Online Supplementary Table 2).”*

Page 10, para 3 to page 11, para 1:

*“…At 17.7 years, the associations were attenuated further with only that between placental area and BA remaining statistically significant (Table 4), albeit with a beta coefficient of similar magnitude to that at earlier timepoints. Associations remained similar in models 2 and 3, but inclusion of child’s height and weight in model 4 removed all relationships. Indeed both placental area and placental volume, adjusted for gestational age at birth, sex and age at the 9 year visit, were weakly correlated with height at 9.9 years (r_partial_ for placental area: 0.08, p=0.02; r_partial_ placental volume: 0.13, p=0.0001). Inclusion of birthweight in the regression analysis also attenuated associations with bone area to below statistical significance (Online Supplementary Table 7). Finally, in conditional analyses in which DXA indices at 17.7 years were controlled for those at 15.5 years (Online Supplementary Table 8), and those at 15.5 years were controlled for those that 9.9 years (Online Supplementary Table 9), in a similar pattern to those with pQCT, all associations were attenuated.”*

Discussion: page 13, para 2 to page 14, para 1:

*“One hypothesis therefore, is that greater placental size leads to earlier onset of puberty, resulting in larger bones at 15.5 years, but with cortical density lagging behind proportionate to bone size (with larger bones at having lower cortical density compared with smaller bones). Indeed, such a mechanism was proposed in a recent study from ALSPAC cohort (**21**), based on all children who underwent pQCT at 15.5 and 17.7 years, linking birthweight to bone outcomes. Here, relationships between birthweight and pQCT measures were somewhat attenuated by adjustment for puberty, and those with cortical density were not apparent at 17.7 years. We found that associations between placental size and pQCT measures at 15.5 years were not appreciably changed by adjustment for puberty; however relationships between placental size and pQCT measures at 17.7 years, whilst robust for periosteal and endosteal circumference, were much weaker for cortical density, consistent with a maturational etiology, and further supported by the conditional models, demonstrating that the strongest placental associations with the earlier time points of follow-up. Conversely, whilst increasing pubertal stage at 13.5 years was associated with larger bones by pQCT, there was no evidence of placental size having been greater in children who were at a later stage of puberty at 13.5 years. Additionally, increasing pubertal stage at 13.5 years was associated with increasing, rather than decreasing cortical density. It must be noted though that the 2 year interval between pubertal staging and pQCT measures somewhat limits the inferences that can be made. Furthermore, the correlation between birthweight and placental area was 0.4, suggesting much scope for relationships between placental size and outcomes independent of birthweight, consistent with previous documentation of the role of placental size versus function (**22**,**23**). Inclusion of birthweight in the base model removed associations between placental size and DXA bone area, most likely due to the strong association between birthweight and overall size, thus potentially on the causal pathway. In contrast, associations between placental size and pQCT measures of periosteal and endosteal circumference, and cortical density, although attenuated, remained similar to those without the inclusion of birthweight, suggesting relationships over and above those mediated through size at birth. Consistent with these findings, although placental size was weakly correlated with height in childhood, and whilst the DXA associations were removed by addition of height in the models, those with the pQCT indices remained statistically significant, further supporting the notion that the placenta pQCT relationships were not purely mediated via linear growth.”*

**4. Page 7: In the second model were the covariates maternal age, maternal height and maternal weight? Or child height and child weight. The description of these models was confusing. What causal pathway and why include each covariate separately? Where in the results are these models referred to? The relationship between placental size and longitudinal growth will be key to the interpretation of this work and it isn't easy to determine whether this has sufficiently been accounted for in the analysis.**

**Page 9, line 27: What is the fully adjusted model, model 3?**

Again we apologise that this was not clearly articulated and have thoroughly revised the statistical methods such that these points are much more easily assimilated.

Methods: page 7, para 1 to page 8, para 1:

*“Statistical analysis*

*All variables were checked for normality. Sex differences between baseline characteristics were compared using unpaired t-tests and chi-squared tests. Pubertal stage information was missing in 100 individuals and in these cases data were imputed: Individuals who did not have pubertal stage information were assigned a value of 4.5, which was close to the mean value (4.46) and stands midway between the two most commonly observed tanner stages- 4 and 5. A sensitivity analysis was undertaken using the complete case data. Univariate and multivariate linear regression were used to relate placental measurements to offspring DXA and pQCT measurements. Although not necessarily true confounders, gestational age is strongly associated with placental size, and offspring age and sex are strongly associated with the bone outcomes: We therefore included these as covariates in all models in order to increase the precision of our estimates. We hypothesised that maternal factors, which have previously been associated with offspring bone mass and body composition, might act through placental size and thus incorporated these into a second model. Puberty has a marked impact on bone development and in a third model we included the child’s pubertal stage at 13.5 years to investigate whether relationships might be mediated via altered timing of pubertal transition. Finally, in order to assess the contribution of the child’s current body size, in a further model we additionally included child’s height and weight at the relevant assessment. The covariates included in the 4 models are summarised below:*

*Model 1: Child’s gestational age at delivery, age at pQCT and sex*

*Model 2: As model 1 and maternal age at delivery, height, weight and parity*

*Model 3: As model 2 and child’s pubertal stage at 13.5 years*

*Model 4: As model 3 and child’s height and weight at 15.5 years*

*In order to assess the contribution of placental size to change in bone outcomes, we used a conditional multiple regression analysis, in which the 4 models were used as before, but for each pQCT or DXA measure, we additionally included the corresponding measure at the previous time point. This effectively yields the association between placental size and the bone measure at a later time point, over and above that explained by the association between placental size and the bone measure at the earlier time point. We undertook separate analyses to assess whether associations might be mediated via birthweight and by including birthweight in model 1. Placental measurements and sex interactions were examined, however these provided little evidence of sex differences and we therefore analysed boys and girls together. In line with convention, DXA-derived whole body bone variables were analysed minus head. To enable comparison of effect sizes across relationships, all predictor and outcome variables were standardised to z-scores with a mean of 0 and an SD of 1. Regression coefficients are therefore representative of SD change in outcome per unit SD change in predictor, and may be interpreted as partial correlation coefficients. Analysis was performed using SPSS Version 21 (IBM UK Ltd, Portsmouth, UK).”*

**Page 9, line 46: Which model attenuated the DXA findings?**

This is an important point, and we have clarified this in the results and in the legend to the modified table 4, which now includes DXA outcomes at 17.7 years.

Page 10, para 3 to page 11, para 1:

*“Placental size and offspring DXA measurements of bone mass*

*At age 9.9 years, positive relationships were observed between each of placental area and volume, and offspring WB (minus head) BA and WB (minus head) BMC (Table 4), similar in boys and girls. No associations between placental measures and child BMD (minus head) were seen. At 15.5 years, similar positive associations between placental area or volume and DXA BA, but those with BMC were partially attenuated (p=0.07 and 0.09 respectively). At 17.7 years, the associations were attenuated further with only that between placental area and BA remaining statistically significant (Table 4), albeit with a beta coefficient of similar magnitude to that at earlier timepoints. Associations remained similar in models 2 and 3, but inclusion of child’s height and weight in model 4 removed all relationships. Inclusion of birthweight in the regression analysis also attenuated associations with bone area to below statistical significance (Online Supplementary Table 7). Finally, in conditional analyses in which DXA indices at 17.7 years were controlled for those at 15.5 years (Online Supplementary Table 8), and those at 15.5 years were controlled for those that 9.9 years (Online Supplementary Table 9), in a similar pattern to those with pQCT, all associations were attenuated.”*

**Minor comments:**

**Page 6 - Include a reference for the pQCT threshold as it isn't a standard one.**

Thank you-we have now included a reference as requested.

**Page 9, line 19 -A later stage of puberty would be better than 'greater'**

We agree with this point and have changed the wording as suggested.

**Reviewer: 2**

**Comments to the Author**

**This is an interesting paper as it focuses on the role of the size of placenta in determining aspects of future health.**

**Fetal growth, most commonly measured as birthweight, is highly dependent on placental function. However, there are no presise measurements of placental function. It would therefore have been interesting to know how the authors have considered the relationship between placental size and function.**

**Since the correlations between placental size and birthweight are strong, it would have been interesting to know how fetal-placental ratio is associated to the outcomes in this paper. It is not clear whether the observed associations between placental size and volume and outcomes also can be explained by birthweight.**

These are important points, and we have incorporated additional models with inclusion of birthweight. These are presented in supplementary tables.

Methods: page 8, para 1:

*“...We undertook separate analyses to assess whether associations might be mediated via birthweight and by including birthweight in model 1. Placental measurements and sex interactions were examined, however these provided little evidence of sex differences and we therefore analysed boys and girls together. In line with convention, DXA-derived whole body bone variables were analysed minus head. To enable comparison of effect sizes across relationships, all predictor and outcome variables were standardised to z-scores with a mean of 0 and an SD of 1. Regression coefficients are therefore representative of SD change in outcome per unit SD change in predictor, and may be interpreted as partial correlation coefficients. Analysis was performed using SPSS Version 21 (IBM UK Ltd, Portsmouth, UK).”*

Results: page 9, para 1 to page 11, para 1:

*“Placental size and offspring pQCT indices at age 15.5 years*

*Table 2 summarises the relationships observed between placental measurements and offspring bone mass. We observed strong positive relationships between placental area and child’s cortical area, periosteal circumference and endosteal circumference at age 15.5 years, which remained robust after adjusting for gestational age, age at pQCT and sex (all p<0.05). Conversely there was a negative association between placental area and cortical BMD [β= -0.1 (95%CI: -0.19, -0.02); p=0.01]. These relationships remained, but were attenuated, after additional adjustments for maternal age at delivery, maternal parity, height and weight, and also after inclusion of child’s pubertal stage at 13.5 years, (except for placental area and cortical area, p=0.06; Table 2, Figure 2). Adjustment for child’s height and weight at 15.5 years did not materially alter the associations observed. Inclusion of birthweight modestly attenuated the beta coefficients, but the overall pattern of associations remained similar (Online Supplementary Table 2).*

*The strongest observed relationships were between placental area and measurements of endosteal and periosteal circumference [EC:β= 0.21 (95%CI: 0.13, 0.30); PC: β= 0.19 (95%CI: 0.10, 0.27)]. Similar relationships were observed between placental volume and child pQCT measurements. There was no association between placental size and cortical thickness; a weak association was observed between placental volume and cortical content, however this relationship was no longer present after maternal and pubertal covariates were incorporated into the regression model. When examined separately by offspring sex, relationships appeared similar in boys and girls, with the p-value for the interaction placental size*sex on pQCT outcomes >0.05. In a sensitivity analysis using the complete case data, results were not materially different from those using imputed values where pubertal status was missing.*

*Online Supplementary Table 3 demonstrates that mean placental area and volume did not differ by pubertal stage at 13.5 years. Online supplementary Table 4 similarly summarises the mean pQCT indices (represented as SD scores) by pubertal status at 13.5 years. Here, there was a trend for greater cortical area, thickness, content and density with later pubertal stage, both in boys and girls (p≤0.01).*

*Placental size and offspring pQCT indices at age 17.7 years*

*Table 3 summarises the relationships observed between placental measurements and offspring bone mass at age 17.7 years. Although the previously observed associations were attenuated, in the fully adjusted models, positive relationships remained between placental size (area and volume) and endosteal circumference and periosteal circumference. The negative association between placental volume and cortical density remained, and that between placental area and cortical density was partially attenuated (p=0.18). Relationships were similar in boys and girls. Again, inclusion of birthweight modestly attenuated the beta coefficients, but the overall pattern of associations remained similar (Online Supplementary Table 5). In a further analysis, in which pQCT indices at age 17.7 years were conditioned on those at 15.5 years, relationships were further attenuated (Online Supplementary Table 6).*

*Placental size and offspring DXA measurements of bone mass*

*At age 9.9 years, positive relationships were observed between each of placental area and volume, and offspring WB (minus head) BA and WB (minus head) BMC (Table 4), similar in boys and girls. No associations between placental measures and child BMD (minus head) were seen. At 15.5 years, similar positive associations between placental area or volume and DXA BA, but those with BMC were partially attenuated (p=0.07 and 0.09 respectively). At 17.7 years, the associations were attenuated further with only that between placental area and BA remaining statistically significant (Table 4), albeit with a beta coefficient of similar magnitude to that at earlier timepoints. Associations remained similar in models 2 and 3, but inclusion of child’s height and weight in model 4 removed all relationships. Inclusion of birthweight in the regression analysis also attenuated associations with bone area to below statistical significance (Online Supplementary Table 7). Finally, in conditional analyses in which DXA indices at 17.7 years were controlled for those at 15.5 years (Online Supplementary Table 8), and those at 15.5 years were controlled for those that 9.9 years (Online Supplementary Table 9), in a similar pattern to those with pQCT, all associations were attenuated.”*

Discussion: page 13, para 2 to page 14, para 1:

*“One hypothesis therefore, is that greater placental size leads to earlier onset of puberty, resulting in larger bones at 15.5 years, but with cortical density lagging behind proportionate to bone size (with larger bones at having lower cortical density compared with smaller bones). Indeed, such a mechanism was proposed in a recent study from ALSPAC cohort (**21**), based on all children who underwent pQCT at 15.5 and 17.7 years, linking birthweight to bone outcomes. Here, relationships between birthweight and pQCT measures were somewhat attenuated by adjustment for puberty, and those with cortical density were not apparent at 17.7 years. We found that associations between placental size and pQCT measures at 15.5 years were not appreciably changed by adjustment for puberty; however relationships between placental size and pQCT measures at 17.7 years, whilst robust for periosteal and endosteal circumference, were much weaker for cortical density, consistent with a maturational etiology, and further supported by the conditional models, demonstrating that the strongest placental associations with the earlier time points of follow-up. Conversely, whilst increasing pubertal stage at 13.5 years was associated with larger bones by pQCT, there was no evidence of placental size having been greater in children who were at a later stage of puberty at 13.5 years. Additionally, increasing pubertal stage at 13.5 years was associated with increasing, rather than decreasing cortical density. It must be noted though that the 2 year interval between pubertal staging and pQCT measures somewhat limits the inferences that can be made. Furthermore, the correlation between birthweight and placental area was 0.4, suggesting much scope for relationships between placental size and outcomes independent of birthweight, consistent with previous documentation of the role of placental size versus function (**22**,**23**). Inclusion of birthweight in the base model removed associations between placental size and DXA bone area, most likely due to the strong association between birthweight and overall size, thus potentially on the causal pathway. In contrast, associations between placental size and pQCT measures of periosteal and endosteal circumference, and cortical density, although attenuated, remained similar to those without the inclusion of birthweight, suggesting relationships over and above those mediated through size at birth. Consistent with these findings, although placental size was weakly correlated with height in childhood, and whilst the DXA associations were removed by addition of height in the models, those with the pQCT indices remained statistically significant, further supporting the notion that the placenta pQCT relationships were not purely mediated via linear growth.”*

**Fetal-placental ratio differs between genders, illustrating that certain aspects of placental functions are sex specific, even if measures of placental size do not differ.**

We agree that sexual dimorphism is important to explore. We have clarified that relationships were similar in boys and girls and that there was no statistically significant interaction between sex and placenta-bone relationships.

Results: page 9, para 2:

*“The strongest observed relationships were between placental area and measurements of endosteal and periosteal circumference [EC:β= 0.21 (95%CI: 0.13, 0.30); PC: β= 0.19 (95%CI: 0.10, 0.27)]. Similar relationships were observed between placental volume and child pQCT measurements. There was no association between placental size and cortical thickness; a weak association was observed between placental volume and cortical content, however this relationship was no longer present after maternal and pubertal covariates were incorporated into the regression model. When examined separately by offspring sex, relationships appeared similar in boys and girls, with the p-value for the interaction placental size*sex on pQCT outcomes >0.05. In a sensitivity analysis using the complete case data, results were not materially different from those using imputed values where pubertal status was missing.”*

Page 10, para 2:

*“Table 3 summarises the relationships observed between placental measurements and offspring bone mass at age 17.7 years. Although the previously observed associations were attenuated, in the fully adjusted models, positive relationships remained between placental size (area and volume) and endosteal circumference and periosteal circumference. The negative association between placental volume and cortical density remained, and that between placental area and cortical density was partially attenuated (p=0.18). Relationships were similar in boys and girls. Again, inclusion of birthweight modestly attenuated the beta coefficients, but the overall pattern of associations remained similar (Online Supplementary Table 5). In a further analysis, in which pQCT indices at age 17.7 years were conditioned on those at 15.5 years, relationships were further attenuated (Online Supplementary Table 6).”*

Page 10, para 3:

*“Placental size and offspring DXA measurements of bone mass*

*At age 9.9 years, positive relationships were observed between each of placental area and volume, and offspring WB (minus head) BA and WB (minus head) BMC (Table 4), similar in boys and girls. No associations between placental measures and child BMD (minus head) were seen....”*

**It would have been useful to include a flowchart of the population included to illustrate the subcohorts undergoing various measurements at different points of time.**

This is a great suggestion and we have incorporated this in the supplementary material.

**Minor comments:**

**Table 1; placental volume is included both under child characteristics and placental characteristics**

Thank you very much for pointing this out. We have corrected the table.

I do hope that this manuscript will now be suitable for publication in the Journal of Bone and Mineral Research, and I look forward to hearing from you in due course.

With all best wishes

Yours sincerely,

**Nicholas C W Harvey, MA MB BChir PhD FRCP**
Professor of Rheumatology and Clinical Epidemiology
